# Prognostic and predictive determinants in high-grade gliomas: integrating tumor-intrinsic biology with patient and system-level factors

**DOI:** 10.3389/fneur.2025.1664458

**Published:** 2025-11-19

**Authors:** William Davalan, Ryan Alkins

**Affiliations:** 1 Faculty of Medicine, McGill University, Montreal, QC, Canada; 2 Division of Neurosurgery, Department of Surgery, Queen’s University, Kingston, ON, Canada

**Keywords:** high-grade glioma, adult-type diffuse gliomas, prognosis, molecular markers, tumor microenvironment, clinical factors, socioeconomic factors, treatment strategies

## Abstract

Adult-type high-grade gliomas (HGGs) represent a biologically heterogeneous and clinically aggressive class of primary central nervous system tumors, characterized by diffuse infiltration, therapeutic resistance, and poor prognosis. Contemporary advances in molecular neuro-oncology have redefined prognostic stratification, shifting from purely histopathological frameworks to integrated molecular classification. This narrative review critically examines the intrinsic biological determinants of prognosis in HGGs, as delineated in the 2021 World Health Organization Classification of Tumors of the Central Nervous System, which differentiates glioblastoma (IDH-wildtype), astrocytoma (IDH-mutant), and oligodendroglioma (IDH-mutant, 1p/19q-codeleted) based on distinct molecular signatures. We examine the prognostic and therapeutic relevance of canonical biomarkers, alongside emerging molecular alterations and autophagy-related gene expression. In addition, we explore the tumor microenvironment and immune landscape of HGGs, and highlight the growing role of radiogenomics and artificial intelligence in integrating imaging with multi-omics data for personalized risk stratification. Beyond tumor-intrinsic biology, increasing attention is being directed toward patient-level and system-level determinants that shape prognosis. This review also synthesizes current evidence on the impact of demographic, clinical, therapeutic, and socio-economic factors influencing survival in patients with HGGs. A multidimensional approach to prognostication that integrates molecular, clinical, and contextual data is therefore essential for both improving survival and advancing health equity. By synthesizing established and emerging prognostic insights, this review underscores the critical role of tumor-intrinsic biology in guiding precision oncology approaches and developing biologically informed prognostic frameworks for patients with HGGs, while supporting the integration of non-biological determinants into clinical frameworks.

## Highlights

- 2021 WHO CNS classifies HGGs into IDH-wildtype glioblastoma, IDH-mutant astrocytoma, and IDH-mutant, 1p/19q-codeleted oligodendroglioma, enabling molecularly driven prognostication.
- Immune and genetic features present targets for precision therapy.
- Patient-specific factors, treatment delivery, and systemic health disparities significantly influence outcomes in high-grade gliomas.
- Integrating clinical, biological, and contextual data is essential for advancing equitable, personalized care.

## 1 Introduction

**Figure 1:**
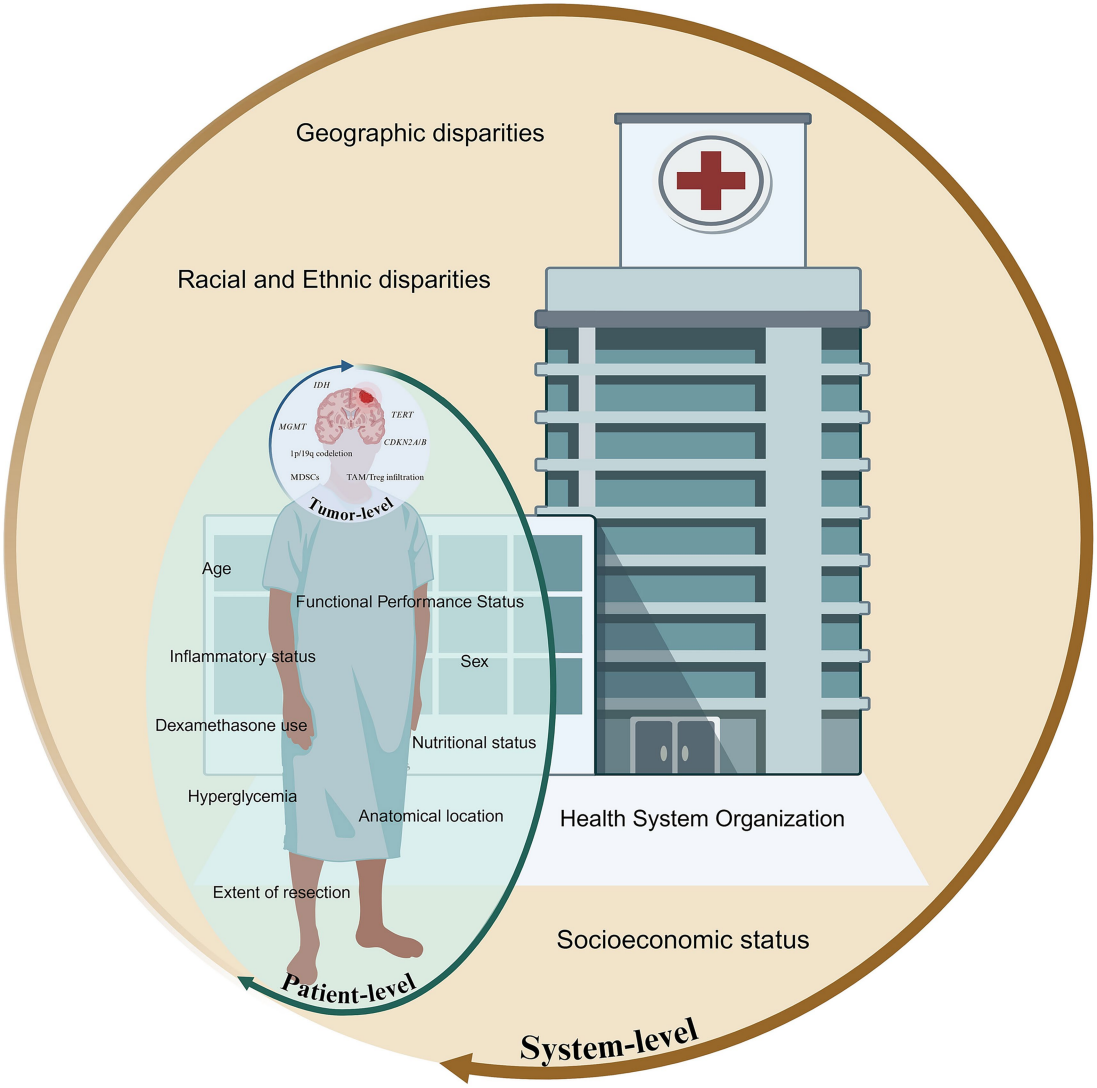
Multiscale framework illustrating tumor, patient, and systemic factors influencing prognosis in high-grade gliomas.

Adult-type diffuse high-grade gliomas (HGGs) are an aggressive group of infiltrative brain tumors, originating from glial cells, including astrocytes, oligodendrocytes, and ependymal cells. Classified as World Health Organization Classification of Tumors of the Central Nervous System (WHO CNS) grade 3 or 4 (1), these tumors exhibit significant malignancy, high recurrence rates, and poor survival outcomes despite aggressive treatment (2, 3). Survival in HGGs is determined by an interplay of histological, molecular, anatomical, and systemic variables (Figure 1), many of which remain incompletely understood or inadequately incorporated into current clinical paradigms. Accurate prognostication is vital for enabling precise prediction of disease trajectory, guides treatment intensity, and supports the selection of appropriate interventions, ensuring care is tailored to each patient’s unique profile. Prognostic biomarkers provide information about the expected clinical course of a disease independent of therapeutic intervention, whereas predictive biomarkers identify patients most likely to derive benefit from a particular treatment (4). In practice, however, these categories frequently converge, with several biomarkers exhibiting both prognostic and predictive significance.

Tumor-intrinsic factors, including the genetic, epigenetic, transcriptional, and microenvironmental attributes of the neoplasm, serve as primary determinants of tumor biological behavior, therapeutic responsiveness, and overall clinical outcomes. Recent advances in molecular oncology and integrative genomics have enabled a more refined understanding of these tumor-specific determinants, offering novel insights into glioma pathogenesis, progression, and intertumoral heterogeneity. However, prognostic models centered exclusively on tumor-intrinsic features fail to capture the full spectrum of clinical variability observed across patients. Patient-level factors are increasingly recognized as independent prognostic determinants that modulate therapeutic efficacy, treatment tolerability, and overall disease trajectory. Compounding these clinical factors are the pervasive and increasingly documented effects of societal determinants of health, with disparities that persist despite biologically similar disease presentations. As a result, prognosis in HGGs is often shaped as much by systemic and patient-level determinants as by the molecular underpinnings of the tumor itself.

This review synthesizes prognostic and predictive determinants of survival in adult-type HGGs. By integrating insights from histopathology, molecular biology, tumor microenvironment, and structural imaging with host-specific clinical factors, treatment-related variables, systemic physiological modifiers, and healthcare delivery disparities, we propose a multidimensional framework that captures the complex interplay of intrinsic and extrinsic factors shaping HGG outcomes and informing biomarker-driven, equitable therapeutic decision-making.

## 2 Intrinsic tumor biology and molecular determinants

### 2.1 Histopathological and molecular classification

The biological behavior of HGGs was traditionally determined primarily by tumor grade, established by histopathological evaluation. HGGs, particularly grade 4 tumors, are characterized by aggressive histological features, including marked nuclear atypia, frequent mitotic activity, neo-angiogenesis, and necrosis. These morphological hallmarks reflect the biological aggressiveness of the tumor, exhibiting rapid growth, extensive invasiveness, and poor clinical outcomes. Histopathological assessment now represents just one element within the more comprehensive tumor classification framework. Earlier editions of the WHO CNS stratified gliomas into types and grades (1) based on tumor architecture and cellular features. The substantial molecular and clinical heterogeneity observed within the same grade and type led to the integration of molecular diagnostics into the 2016 WHO CNS (5). This update introduced key biomarkers, including1p/19q codeletion for diagnosing oligodendroglioma, and *IDH* mutation status, along with several other molecular markers and histologic features, to differentiate astrocytoma subtypes. Since then, the adoption of advanced technologies (6) such as next-generation sequencing, RNA expression profiling, and DNA methylation profiling have enabled the discovery and classification of new entities, as well as more precise stratification of existing tumors, culminating in the most recent 2021 WHO CNS fifth edition (1) (2021 WHO CNS5). This revised 2021 WHO CNS5 tumor classification system marked a paradigm shift by fully integrating molecular features into glioma classification and grading, combining traditional histopathological evaluation with key molecular markers (e.g., *IDH* mutation status, 1p/19q codeletion, *CDKN2A/B* deletions, and histone mutations) to more accurately reflect the biological prognosis, and predictive therapeutic implications of these tumors (1) (Table 1). It introduces the category of adult-type diffuse gliomas, which is divided into three distinct molecularly defined subtypes: IDH-wildtype Glioblastoma, IDH-mutant Astrocytoma, IDH-mutant and 1p/19q-codeleted Oligodendroglioma.

**Table 1:** The 2021World Health Organization (WHO) central nervous system classification of adult high-grade gliomas.

Features	Tumor types		
	Glioblastoma	Astrocytoma	Oligodendroglioma
Diagnostic markers	*IDH-wildtype*	*IDH-mutant*	*IDH-mutant, 1p/19q co-deleted*
WHO grade	4	3–4	3
Molecular markers	*TERT* promoter mutation *EGFR* amplification *PTEN* loss +7/−10 chromosomal alteration *MGMT* promoter methylation PI3K, p53, CDK4/6 pathways	*IDH1*/*IDH2* mutation *ATRX* loss *TP53* mutation *CDKN2A*/*B* deletion (grade 4)	*IDH1*/*IDH2* mutation 1p/19q co-deletion *TERT* promoter mutation *CIC* *NOTCH1* *FUBP1*
Histopathology	Highly cellular, pleomorphic, necrosis, pseudopalisading, microvascular proliferation	Increased cellularity, nuclear atypia, mitotic activity, necrosis in grade 4	Round, uniform nuclei, perinuclear halos (“Fried-egg”) cells, delicate capillary network, calcifications
Median survival	Poor (~12–18 months)	Intermediate (~3–7 years)	Best among HGGs (~10 + years)

### 2.2 Established molecular prognostic and predictive biomarkers by glioma subtype

#### 2.2.1 IDH-wildtype glioblastoma

At the most aggressive end of the glioma spectrum is IDH-wildtype glioblastoma, now strictly defined under 2021 WHO CNS5 as grade 4 (1) (Table 1). The methylation status of the O^6^-methylguanine-DNA methyltransferase (*MGMT*) promoter methylation represents a well-established molecular biomarker for prognosis and a predictive marker of response to alkylating therapy in IDH-wildtype glioblastoma. *MGMT*, located on chromosome 10q26.3, encodes a DNA repair enzyme responsible for the removal of alkyl groups from the O^6^ position of guanine, a primary site of damage induced by alkylating chemotherapeutic agents such as temozolomide (TMZ) and nitrosourea derivatives. Methylation of the *MGMT* promoter (*MGMTp*) leads to transcriptional silencing, resulting in impaired DNA repair mechanisms and, consequently, increased tumor sensitivity to alkylating chemotherapy. The pivotal study by Hegi et al. (2005) (7), conducted within the landmark EORTC–NCIC phase III trial comparing radiotherapy alone to radiotherapy with TMZ (the Stupp protocol), established *MGMT* promoter methylation as both a prognostic and predictive biomarker in glioblastoma. Among 206 cases, *MGMT* promoter methylation was present in 45% and independently associated with longer overall survival (*p* < 0.001). Patients with methylated tumors showed a marked benefit from TMZ, with overall survival (OS) and progression-free survival (PFS) of 21.7 and 10.3 months, respectively, versus 15.3 and 5.9 months with radiotherapy alone (*p* = 0.007 and *p* = 0.001). In contrast, unmethylated tumors derived minimal benefit (median OS 12.7 vs. 11.8 months; *p* = 0.06), underscoring the predictive value of *MGMT* methylation for alkylating chemotherapy response. More recently, in a cohort of 111 IDH-wildtype glioblastoma patients (8) who underwent gross total resection (GTR) followed by standard Stupp protocol treatment (maximal safe surgical resection followed by radiation therapy and TMZ), *MGMT* methylation status was shown to be significantly associated with improved PFS and OS (*Unmethylated*: PFS 7.2 months, OS 13.4 months; *Low methylation* [10–20%]: PFS 10.4 months, OS 17.9 months; *High methylation* [>20%]: PFS 19.83 months, OS 29.93 months; *p* < 0.05). Importantly, *MGMT* promoter methylation serves as a clinically significant predictive biomarker in the treatment planning of elderly glioblastoma patients, guiding therapeutic decisions and predicting response to TMZ-based therapy. Randomized trials (9–11) have demonstrated that elderly patients with *MGMT*-methylated (IDH-non-specified) glioblastoma achieve superior survival with TMZ alone compared to radiotherapy alone without increasing toxicity. In contrast, patients with truly *MGMT* unmethylated tumors derived no benefit from TMZ treatment (12), with radiotherapy alone offering more favorable outcomes in this subgroup. In older populations, where chemotherapy-related toxicity may be more of a concern, *MGMT* methylation status helps identify patients most likely to benefit from TMZ, facilitating a personalized approach that optimizes therapeutic efficacy while minimizing treatment burden. In select cases of elderly or poor-performance-status patients with *MGMT*-methylated glioblastoma, TMZ monotherapy has demonstrated comparable efficacy to radiotherapy (9, 11), offering a tolerable and effective alternative when radiation is contraindicated or declined. Consequently, the 2017 *European Association for Neuro-Oncology* (EANO) guidelines (13) recommend *MGMT* testing as standard practice in elderly patients (>65–70 years), advising TMZ-based therapy for *MGMT*-methylated tumors and hypofractionated radiotherapy alone for unmethylated cases. Beyond its established predictive value for alkylating chemotherapy, *MGMT* promoter methylation also appears to modulate outcomes in the era of adjunctive therapies. In the pivotal EF-14 trial evaluating Tumor-Treating Fields (TTFields) combined with maintenance TMZ, survival benefits were observed across molecular subtypes regardless of *MGMT* status, but patients with *MGMT*-methylated tumors experienced the most pronounced gains (14), highlighting that *MGMT* methylation retains prognostic relevance even in the context of adjunctive therapies (15). Further efforts to improve chemotherapy efficacy include the addition of Lomustine to TMZ in patients with *MGMT*-methylated glioblastoma, resulting in median survival benefits of 48.1 months versus 31.4 months in a recent phase III clinical trial (16), albeit with increased toxicity. However, emerging evidence suggests that the predictive value of *MGMT* methylation in glioblastoma is not uniform but is modulated by broader epigenetic subgroups and degrees of methylation. The NOA-08 trial demonstrated that TMZ provided the greatest benefit in tumors classified under the “receptor tyrosine kinase (RTK) II” methylation subtype, indicating that *MGMT* methylation status interacts with distinct epigenetic landscapes (17). Additionally, recent studies exploring *MGMT* methylation hotspots and quantitative methylation thresholds suggest that the degree of methylation may further refine its predictive utility, distinguishing partial responders from true non-responders (18). Additional factors, such as Telomerase Reverse Transcriptase (*TERT*) promoter mutations, may further modulate its predictive power, emphasizing the need for a more comprehensive biomarker-driven approach to glioblastoma treatment.

The *TERT* gene is an important component of the telomerase complex, responsible for maintaining telomere length and preventing chromosomal instability. *TERT* promoter mutations (*TERTp*) occurs in approximately 80% of IDH-wildtype glioblastomas (19, 20) and enable tumor cells to evade replicative senescence, contributing to the malignant phenotype. These mutations are now integrated into the 2021 WHO CNS5 classification as key molecular markers for IDH-wildtype glioblastomas and oligodendrogliomas (1). Despite their high prevalence, the prognostic significance of *TERTp* mutations remains a subject of debate. Some studies (20–23) associate *TERTp* mutations with poor survival outcomes, while others (19, 24, 25) suggest that their clinical impact is context-dependent, particularly in relation to *MGMT* methylation status. A retrospective study (26) of 453 IDH-wildtype glioblastoma patients found that tumors harboring both *MGMT* methylation and *TERT* mutations exhibited the longest survival, whereas *MGMT*-unmethylated/*TERT*-mutated tumors had the poorest outcomes, suggesting a potential synergistic interaction between these molecular alterations. However, another study (25) found no consistent evidence that *TERT* mutations enhance the survival benefit of *MGMT* methylation.

Beyond *MGMT* and *TERT* alterations, chromosomal aberrations are hallmark features of IDH-wildtype glioblastoma, particularly chromosome 7 gain (+7) and chromosome 10 loss (−10), which contribute to genomic instability and tumor aggressiveness (27). These alterations frequently coincide with epidermal growth factor receptor (*EGFR*) amplification, a major oncogenic driver observed in approximately 40–50% of cases (28). *EGFR* dysregulation, especially via amplification or constitutively active variants like *EGFRvIII*, enhances tumor proliferation, invasiveness, and resistance to standard therapies, including radiotherapy and TMZ. These genomic alterations are strongly associated with poor OS and PFS (28), underscoring their prognostic significance and their role in the molecular classification of glioblastoma. Despite extensive efforts, *EGFR*-targeted therapies (e.g., tyrosine kinase inhibitors, monoclonal antibodies, and vaccine-based approaches like rindopepimut) have shown limited efficacy in clinical trials, largely due to challenges in BBB penetration and intrinsic tumor resistance (29). Another key consequence of chromosome 10 loss is the deletion or inactivation of *PTEN*, leading to *PI3K/AKT/mTOR* pathway hyperactivation, which sustains tumor cell survival and proliferation. *PTEN* mutations occur in approximately 40% of cases and are associated with poor prognosis and therapy resistance (30).

#### 2.2.2 IDH-mutant astrocytomas

IDH-mutant diffuse astrocytomas are defined by the 2021 WHO CNS5 classification as tumors harboring a gain-of-function mutation in *IDH1* or *IDH2* and are graded from 2 to 4 based on histopathologic features, including anaplasia, mitotic activity, necrosis, microvascular proliferation, and homozygous *CDKN2A/B* deletion (1). Tumor grading plays a significant role for IDH-mutant diffuse astrocytomas prognosis with grade 3 tumors exhibiting a median OS of 8.1 years, whereas grade 4 tumors have a significantly shorter median OS of 4.7 years (*p* < 0.05) (31, 32). A critical prognostic factor in IDH-mutant diffuse astrocytomas is the presence of *CDKN2A/B* deletions (1, 32), which are strongly associated with worse survival outcomes. Patients with *CDKN2A/B* deletions exhibit a median OS of 1.8 years, compared to 5.5 years (*p* < 0.001) in those without the deletion (32).

A major shift in the 2021 WHO CNS5 classification was the differentiation of glioblastomas from astrocytomas based on *IDH* status. *IDH1/2* mutations are typically single-point mutations that result in a neomorphic enzyme activity, converting *α*-ketoglutarate (α-KG) to D-2-hydroxyglutarate (2-HG), an oncometabolite (33). The accumulation of 2-HG disrupts cellular metabolism and leads to widespread DNA and histone hypermethylation, creating an altered epigenetic landscape that promotes tumorigenesis (33). The presence of *IDH* mutations is now a firmly established favorable prognostic factor in diffuse astrocytomas and oligodendrogliomas. While the *IDH1^R132H^* mutation is the most common, accounting for over 90% of IDH-mutant gliomas, non-canonical *IDH1* mutations have also been associated with improved survival outcomes (34). In a cohort of 433 patients with *IDH1*-mutated grade 2–3 gliomas (34), approximately 9.9% had non-canonical mutations, and presented with younger age at diagnosis and favorable prognosis (198.6 months vs. 138.5 months; *p* < 0.05). This was supported by an analysis of the CATNON trial’s 1p/19q non-codeleted astrocytoma samples that showed that patients with non-R132H mutations had better outcomes, attributable to higher levels of genome-wide DNA methylation (35). These tumors predominantly affect younger individuals, with an approximated median age of 35 (32, 36, 37). Beyond prognosis, *IDH* mutations also serve a predictive role, indicating potential responsiveness to IDH inhibitors. Phase I and II (38–40) trials have demonstrated that these inhibitors exhibit the greatest efficacy in non-enhancing gliomas, particularly in grade 2 and 3 tumors, where longer PFS has been observed compared to enhancing tumors (39).

While *MGMTp* methylation has long been established as a favorable prognostic and predictive marker in IDH-wildtype glioblastomas, its role in IDH-mutant gliomas has been more nuanced. Recent evidence shows that in IDH-mutant gliomas, *MGMTp* methylation is significantly more prevalent and is independently associated with improved OS and PFS when treated with TMZ (41). However, its predictive value appears restricted primarily to grade 4 tumors, with no consistent predictive utility in lower-grade IDH-mutant astrocytomas (41). Furthermore, *MGMT* methylation in IDH-mutant astrocytomas may interact with other epigenetic modifiers, particularly *PRMT5*, to modulate tumor progression and therapeutic response (42). Notably, IDH-mutant astrocytomas exhibiting both *MGMTp* methylation and elevated *PRMT5* expression show significantly prolonged PFS with TMZ monotherapy compared to other subgroups (42). These findings underscore the need for a more nuanced interpretation of *MGMT* methylation, considering both tumor grade and epigenetic context.

Additionally, the majority of IDH-mutant astrocytomas are characterized by *TP53* mutations, often coupled with *ATRX* loss in IDH-mutant gliomas, signal genomic instability and are generally predictive of better outcomes (43, 44). *ATRX* alterations have important biological and therapeutic implications. Loss of *ATRX* impairs replication fork stability and homologous recombination repair, leading to replication stress and activation of the ATR–CHK1 axis. These defects sensitize glioma cells to poly (ADP-ribose) polymerase (PARP) inhibitors, an effect that mirrors the synthetic lethality observed in homologous recombination–deficient tumors. Synergistic vulnerabilities have also been demonstrated with combined PARP and ATR inhibition, further underscoring *ATRX’s* role as a predictive biomarker for DNA damage response–targeted therapies (45–47). In parallel, bioinformatic analyses of TCGA cohorts have shown that ATRX-mutant glioblastomas display higher microsatellite instability and tumor mutational burden, as well as sensitivity to multiple chemotherapeutic and anticancer agents acting on DNA damage pathways, highlighting potential for therapeutic exploitation (48). Clinically, *ATRX* mutations are strongly associated with younger age, co-occurring *IDH* and *TP53* mutations, and prolonged overall survival, reinforcing their dual prognostic and predictive significance in glioma biology (49). The Consortium to Inform Molecular and Practical Approaches to CNS Tumor Taxonomy (cIMPACT-NOW) (50) has proposed a practical diagnostic framework for diffuse gliomas that incorporates ATRX and p53 immunohistochemistry, providing valuable insights into glioma subtyping in both clinical and research settings.

#### 2.2.3 IDH-mutant and 1p/19q-codeleted oligodendroglioma

Oligodendrogliomas have long been associated with a more favorable prognosis, and are defined by the combined presence of *IDH1/2* mutations and 1p/19q codeletion (1). Oligodendrogliomas are relatively indolent gliomas that predominantly affect younger adults, with median survival times exceeding 10 years (51, 52). Current therapeutic guidelines recommend a multimodal approach, consisting of radiation therapy followed by adjuvant chemotherapy with the PCV regimen [Procarbazine, Lomustine (CCNU), and Vincristine] (53). The 1p/19q codeletion is a strong predictive marker of enhanced response to combined radiotherapy and PCV chemotherapy. In the RTOG 9402 trial (53), patients with 1p/19q-codeleted tumors had significantly longer OS, with the combination of PCV chemotherapy and radiotherapy achieving a median OS of 14.7 years compared to 2.7 years in non-codeleted tumors (*p* < 0.001). The EORTC 26951 trial (54) supported the findings that patients with 1p/19q codeleted tumors responded significantly better to combined chemotherapy and radiotherapy, underscoring the favorable prognosis for these tumors. The molecular landscape of oligodendrogliomas includes frequent *TERTp* mutations (55), retained *ATRX* expression, and the absence of p53 accumulation, as 1p/19q codeletion is mutually exclusive with *TP53* and *ATRX* alterations (56, 57). In a subset of cases, deletions in *CDKN2A/B* have been identified; an alteration that is correlated with more aggressive tumor behavior and poorer prognostic outcomes (58).

### 2.3 Tumor microenvironment and immune factors

The tumor microenvironment (TME) plays a central role in shaping the biological behavior and clinical outcomes of HGGs (59). Glioblastoma in particular establishes a profoundly immunosuppressive milieu that promotes tumor progression (59), limits host immune surveillance, and contributes to therapeutic resistance. Distinct transcriptional subtypes (60) (proneural, classical, and mesenchymal) exhibit unique immune signatures, underscoring the heterogeneity of microenvironmental influences on survival (60).

Among the most extensively studied microenvironmental features are tumor-associated macrophages (TAMs), which constitute up to 30–50% of the glioblastoma mass (61). These cells predominantly acquire an M2-like, immunosuppressive phenotype that promotes angiogenesis, extracellular matrix remodeling, and the release of growth factors and cytokines (e.g., IL-10, TGF-*β*) that facilitate immune evasion (62). High TAM density has been consistently correlated with inferior OS (63, 64), particularly in mesenchymal glioblastoma subtypes, where macrophage-related gene expression signatures are enriched. Quantitatively, immunohistochemical studies often categorize “high” TAM infiltration using semi-quantitative bins [e.g., >50 CD163^+^ macrophages per high-power field (HPF) in glioma (65)] and meta-analyses indicate that dichotomized infiltration densities predict survival differences (63). Regulatory T cells (Tregs) represent a second immunosuppressive population that contributes to negative prognosis (66). Elevated Treg infiltration, typically defined as cases with FOXP3^+^ regulatory T cells in the top tertile or above-median infiltration among tumor-infiltrating lymphocytes, has been associated with shorter PFS and OS in glioblastoma patients (67, 68). Similarly, myeloid-derived suppressor cells (MDSCs) accumulate in both peripheral circulation and tumor parenchyma, dampening antigen presentation and cytotoxic activity, with high levels linked to worse outcomes (69). Despite these associations, standardized quantitative thresholds remain lacking across modalities. Most studies rely on relative measures, such as median or quartile stratification, rather than fixed numeric cut-offs (e.g., “≥5% of TILs” or “≥10 cells/HPF”), limiting cross-study comparability. Harmonized quantitative immunoprofiling and standardized scoring systems will be essential to integrate these immune parameters into prognostic and predictive frameworks and to clarify their implications for therapeutic decision-making.

CD13 (aminopeptidase N) has emerged as an additional prognostically relevant microenvironmental marker. Expressed on endothelial cells, pericytes, and subsets of tumor-associated myeloid cells, CD13 regulates extracellular matrix degradation, neoangiogenesis, and cytokine signaling within the glioma TME. Its upregulation promotes tumor vascularization and supports M2-like macrophage polarization, collectively enhancing immune evasion and tumor invasiveness (70). High CD13 expression in glioblastoma has been correlated with increased microvessel density, hypoxia-inducible factor (HIF-1α) activity, and poorer survival (71). Moreover, CD13-positive stromal and immune compartments show enhanced expression of VEGF and IL-6 signaling pathways, underscoring its role as a mediator of the pro-tumorigenic and immunosuppressive milieu.

Immune checkpoint signaling constitutes another axis of prognostic relevance. Upregulation of PD-1, PD-L1, and CTLA-4 within the glioma TME is associated with advanced disease stage and poorer survival, even independent of therapeutic intervention. Although immune checkpoint inhibitors (ICIs) have yielded limited efficacy in clinical trials (72–74), checkpoint expression levels remain biologically meaningful as predictive biomarkers of response and as prognostic markers of an immunosuppressive milieu. For instance, increased PD-L1 and PD-1 expression correlates with more aggressive tumor phenotypes and reduced survival (75–77), although methodological heterogeneity in detection (IHC, RNA-seq, qPCR) has complicated validation. To aid patient stratification, IHC-based scoring systems such as the Tumor Proportion Score (TPS) quantify PD-L1-positive tumor cells, with approximately 61% of gliomas exhibiting at least 1% positivity (75). The Combined Positive Score (CPS), which accounts for PD-L1 expression on both tumor and immune cells, further refines patient selection for immunotherapy. Additionally, circulating PD-1 + T cells, quantified via flow cytometry, correlate with glioma tumor grade, underscoring their prognostic value. Findings from a recent systematic review of the tumor (78)-infiltrating CD8 + T-cell/PD-L1 axis provide further nuance to this relationship. PD-L1 overexpression was consistently associated with inferior OS in glioma patients who had not undergone chemoradiotherapy, whereas its prognostic value diminished or became inconsistent following treatment exposure. Similarly, high infiltration of CD8 + T cells was linked to improved survival in treatment-naïve gliomas but paradoxically predicted worse outcomes after radio/chemotherapy, reflecting therapy-induced T-cell exhaustion and upregulation of inhibitory checkpoints. Importantly, a high PD-1+/CD8 + T-cell ratio was associated with significantly poorer PFS and OS, underscoring that the functional phenotype of infiltrating T cells may be more informative than absolute numbers (78). These findings reinforce that checkpoint expression and immune infiltrates must be interpreted in the context of prior therapy and cellular exhaustion states.

Emerging data further highlight the role of molecularly defined immune signatures in prognostication. CD44 overexpression, frequently associated with mesenchymal transition, enhances TAM recruitment and portends unfavorable outcomes (79). Likewise, elevated expression of MARCO on macrophages identifies mesenchymal glioblastoma with particularly poor prognosis (80). The CD47–SIRPα axis (81), which suppresses phagocytic clearance, has also been linked to diminished survival, while preclinical blockade of this pathway improves anti-tumor immunity. Moreover, transcriptomic profiling has identified high TREM2 expression as a correlate of increased T-cell infiltration, raising the possibility that microenvironmental states defined by this receptor may carry prognostic and predictive implications (82).

### 2.4 Emerging molecular and translational biomarkers

Liquid biopsy, particularly circulating tumor DNA (ctDNA), offers a minimally invasive method for dynamic tumor profiling. ctDNA has been detected in plasma and cerebrospinal fluid of glioma patients, correlating with tumor burden and survival (80). Elevated preoperative ctDNA levels and the detection of somatic mutations have been associated with early progression, and longitudinal ctDNA monitoring can signal recurrence before radiographic progression is evident (81). Additionally, ctDNA may serve as an early predictive biomarker of TMZ resistance, with recent studies identifying mismatch repair pathway mutations in plasma that were absent in the primary tumor but emerged during/after TMZ exposure, suggesting therapy-induced clonal evolution (82). Autophagy-related signatures represent another prognostic and therapeutic avenue. High autophagic activity in glioblastoma contributes to resistance under hypoxic and metabolic stress and is associated with poor survival (83, 84). Expression-based autophagy risk scores can stratify patients, and early-phase trials combining autophagy inhibitors such as hydroxychloroquine with chemoradiation have shown promising results (85), although a separate trial in older patients did not show a clear improvement (86). In parallel, rare but actionable genetic alterations such as BRAF V600E mutations and NTRK fusions have shown significant responsiveness to targeted therapies. The ROAR (Rare Oncology Agnostic Research) basket trial demonstrated that combined dabrafenib and trametinib yielded an objective response rate of 33% and a disease control rate of 85% in BRAF V600E-mutant HGGs patients (87). Similarly, TRK inhibitors such as larotrectinib and entrectinib have yielded high response and disease control rates in NTRK-fused gliomas, despite their low prevalence (88). Collectively, these biomarkers not only refine prognosis but also play a predictive role as they inform targeted treatment strategies, moving HGG management toward a more personalized, biology-driven paradigm.

## 3 Host and system factors

### 3.1 Patient demographics and baseline clinical factors

#### 3.1.1 Age at diagnosis

Age is one of the most extensively studied prognostic factors in gliomas, with its negative impact being particularly pronounced in IDH-wildtype glioblastoma. However, the prognostic significance of age must be interpreted within the framework of molecular classification, as each glioma subtype has a distinct age of onset and disease trajectory. IDH-wildtype glioblastoma is typically diagnosed at a median age of 64 years (83), whereas IDH-mutant astrocytoma and IDH-mutant oligodendroglioma have younger median diagnostic ages of around 35–45 years (84). While survival declines sharply with advancing age in IDH-wildtype glioblastoma, the impact of age in IDH-mutant astrocytomas and oligodendrogliomas is more nuanced, with molecular alterations playing a dominant role in shaping clinical outcomes rather than age alone. For IDH-wildtype glioblastoma, several population-based studies (83, 85) have established age as a strong prognostic determinant, with, survival outcomes decreasing progressively with advancing age, even among patients receiving comprehensive standard-of-care treatment (86). One study reported a median OS of 16.7 months in patients under 50, compared to 5.6 months in those over 70 (*p* < 0.01) (83). This decline in survival may be partly attributed to age-related immune dysregulation, including elements of immunosenescence and altered neuroinflammatory responses within the CNS (87). In contrast, age appears to have a less pronounced prognostic impact in IDH-mutant gliomas (32, 84), with distinctions between IDH-mutant astrocytomas and oligodendrogliomas (88). Most studies investigating IDH-mutant astrocytomas have failed to establish age as a significant prognostic determinant (32). While one study identified an association between age and prognosis exclusively in patients over 60 years with grade 3 astrocytoma, another reported a modest correlation in individuals over 50 years (89), suggesting that age may have a relatively limited role within this molecular subgroup. Conversely, studies (90) examining IDH-mutant, 1p/19q-codeleted oligodendrogliomas have demonstrated a more variable prognostic influence of age. A study found on separate analysis of oligodendroglioma and astrocytoma a significant association with higher age and worse survival in patients with oligodendroglioma but not with astrocytoma (88). Additional insights from the French POLA network suggest that age may influence prognosis at more advanced thresholds, reporting worse outcomes in patients over 70 years diagnosed with IDH-mutant grade 3 and 4 gliomas, the majority of whom had grade 3 IDH-mutant, 1p/19q-codeleted oligodendroglioma (91). These findings indicate that while age is not a major prognostic determinant in IDH-mutant astrocytomas, it may have a more pronounced impact in IDH-mutant oligodendrogliomas. The disparity in the prognostic significance of age between IDH-wildtype glioblastoma and IDH-mutant gliomas is likely driven by the inherently aggressive nature of IDH-wildtype tumors, age-related immune dysfunction, and reduced treatment tolerance to chemotherapy and radiotherapy given the older age at diagnosis for glioblastoma patients, whereas the more indolent course of IDH-mutant gliomas may attenuate the influence of age on prognosis (84). Finally, an important consideration in evaluating the prognostic significance of age in HGGs is the influence of treatment decisions and patient preferences on survival outcomes in elderly patients. Older individuals may be less likely to pursue or be offered aggressive therapy due to concerns regarding treatment-associated toxicity, diminished quality of life (QoL), or preexisting comorbidities, often resulting in lower rates of maximal surgical resection and adjuvant therapy. Historical treatment paradigms have frequently favored less intensive therapeutic approaches for older patients, with some clinicians adopting more conservative surgical and adjuvant strategies based on perceived risks and limited expected benefit. Indeed, older or frail patients are more often treated with hypofractionated radiotherapy schedules to balance efficacy and tolerability (11), potentially contributing to the observed prognostic gradient with age. These factors introduce significant challenges in disentangling the biological impact of age from treatment-related variables in survival analyses, as differences in prognosis may, in part, reflect variations in therapeutic intensity rather than intrinsic tumor aggressiveness. Nonetheless, emerging evidence (92) suggests that carefully selected elderly patients may derive substantial benefits from standard-of-care interventions, reinforcing the necessity of individualized treatment strategies that integrate both patient-specific considerations and evolving clinical evidence.

#### 3.1.2 Performance status

Performance status is a well-established prognostic factor in patients with HGGs, most commonly assessed using the Karnofsky Performance Status (KPS) scale. The KPS provides a standardized, quantitative measure of a patient’s functional capacity and level of independence in daily activities, encompassing a spectrum from full functional autonomy to severe disability (93). However, the KPS has limitations, as it does not comprehensively evaluate cognitive, emotional, or specific neurologic impairments frequently associated with tumor burden. Numerous studies (93–96) have demonstrated that pre-treatment, as well as postoperative performance status, serve as independent prognostic indicators of survival. Kawauchi et al. (95) identified both preoperative and postoperative KPS ≤ 60 as significant predictors of shorter survival. Conversely, Liu et al. (96) reported that a postoperative KPS ≥ 80, along with total resection and adherence to the Stupp protocol, was a strongly associated with improved prognosis. Chambless et al. (93) further highlighted that postoperative KPS score has superior predictive value compared to pre-operative KPS, while Sasaki et al. (94) found that KPS at discharge and the degree of improvement in the KPS between admission and discharge were associated with a favorable prognosis, underscoring the importance of monitoring KPS progression over time. Beyond KPS, additional functional assessment tools, including the *Eastern Cooperative Oncology Group* (ECOG) scale and the *Neurologic Assessment in Neuro-Oncology* (NANO) scale, have been developed and investigated to provide more nuanced evaluations of patient functional status.

Notably, functional performance status is particularly relevant alongside age, with older patients often presenting with lower KPS and ECOG scores. Integrating functional status into treatment planning is essential, as research (92) has suggested that elderly patients who are in good pre- and post-operative condition may achieve survival outcomes comparable to younger patients when managed with multimodal standard care protocols. Thus, KPS remains a pivotal factor in guiding treatment decisions for elderly patients, particularly when evaluating the feasibility of aggressive therapeutic strategies.

#### 3.1.3 Sex

Sex differences in glioblastoma have been consistently documented in epidemiological studies, with a higher incidence observed in males compared to females. The male-to-female incidence ratio is approximately 1.6:1 (97, 98) for glioblastomas; and 1.3:1 for IDH-mutant gliomas (98). While the impact of sex on survival outcomes in other HGGs subtypes remains uncertain, several studies (97) suggest that female patients exhibit superior OS compared to males, although research is scarce. A large-scale analysis utilizing the SEER database (99) demonstrated that female patients had a significantly higher five-year cancer-specific survival rate than males. Differences in tumor localization between sexes have also been reported, with male patients more likely to develop glioblastomas in the frontal lobe, whereas temporal lobe involvement is more common in females (100). Additionally, volumetric analysis (101) has revealed that women tend to present with larger tumors and greater necrotic areas compared to men. Sex-based disparities extend beyond tumor characteristics to treatment approaches and timelines, as studies have reported that a higher percentage of male patients receive multimodal treatment compared to females and that men tend to undergo surgical resection later than women (102). These differences may be influenced by socio-cultural dynamics, including health-seeking behaviors, access to care, and support networks.

Moreover, the underlying biological mechanisms contributing to these sex differences involve a complex interplay of environmental, genetic, immunologic, and hormonal factors. Distinct genetic risk factors have been identified, with *EGFR*-associated risks more prevalent in males, while *TERT*-related risks appear more relevant in females (103). An influence in the proportion of patients with *MGMT* promoter methylation and tumor response to standard treatment (97) in a sex specific manner have also been identified (104). Large-scale molecular profiling from The Cancer Genome Atlas (TCGA) and the Chinese Glioma Genome Atlas (CGGA) datasets (105) has revealed sex-specific DNA methylation and gene expression profiles, highlighting genes such as *NOX*, *FRG1BP*, *AL354714.2*, *PUDP*, *KDM6A*, *DDX3X*, and *SYAP1*, which may contribute to sex-dependent differences in glioblastoma pathogenesis. X-linked tumor suppressor genes further modulate these disparities, particularly *KDM6A*, which escapes X-inactivation and is expressed at higher levels in female cells, enhancing tumor suppression. Beyond genetic predispositions, male astrocytes have been found to exhibit greater susceptibility to malignant transformation, primarily due to intrinsic responses to *TP53* loss (97), which lead to *RB1* downregulation and tumorigenic progression (106). In contrast, female astrocytes were shown to exhibit higher *CDKN1A* expression, even in the presence of *TP53* dysfunction, thereby enforcing stronger cell cycle regulation and reducing transformation susceptibility (107). Hormonal influences also contribute to sex-based differences in glioblastoma progression and prognosis. Experimental models (108) support a protective role of estrogen, with studies demonstrating that estrogen administration improves survival in glioblastoma models. Increased estrogen receptor methylation in glioblastoma tumors suggests a potential tumor-suppressive function of estrogen signaling. Furthermore, studies (109) have reported that higher estrogen receptor and aromatase expression levels correlate with prolonged survival and reduced tumor viability following estradiol treatment, with isoform-specific implications for prognosis. In contrast, androgen receptor signaling in males has been implicated in glioblastoma progression, with evidence (110) suggesting that androgen receptor activation promotes tumorigenesis, potentially by inhibiting tumor-suppressive TGF-*β* signaling. These findings underscore the importance of incorporating sex-specific molecular data into glioblastoma prognostication and therapeutic strategies.

#### 3.1.4 Other clinical factors

The presence and severity of comorbidities are increasingly recognized as influential factors in the prognosis of glioma patients. Studies indicate that patients with high Charlson Comorbidity Index (CCI) scores tend to have significantly shorter OS (111) as comorbid conditions, such as cardiovascular disease, diabetes, hypertension, and chronic respiratory issues, can limit treatment options and reducing the patient’s ability to tolerate aggressive therapies. Clinicians often balance the risks posed by existing health conditions against the benefits of aggressive glioma treatments, with an emphasis on quality of life, particularly in elderly or frail patients. A systematic review by Yoshikawa et al. (112) examining modifiable risk factors revealed that higher body mass index (BMI), alcohol consumption, and NSAID use demonstrated a protective effect against developing glioblastoma.

### 3.2 Surgical and anatomical determinants

#### 3.2.1 Extent of resection and residual tumor volume

Surgical resection of HGGs serves three principal roles: relieving mass effect to provide symptomatic improvement, obtaining tissue for histopathological and molecular characterization, and achieving cytoreduction to minimize therapy-resistant clones. Due to their highly infiltrative nature, HGGs are never completely resected, with this being evident from the low survival rates, and recurrences within 2 cm of the resection margins (113). The extent of resection (EOR) is a well-established prognostic factor in HGG treatment and is evaluated by contrast-enhanced MRI 24 to 48 h after surgery. Traditionally, EOR has been quantified as the percentage reduction in preoperative tumor volume, with gross total resection (GTR), defined as complete removal of all visible enhancing tumor on postoperative imaging, generally associated with better survival outcomes compared to subtotal resection (STR) or biopsy. In an effort to standardize classification, the *Response Assessment in Neuro-Oncology* (RANO) group has further refined EOR classifications into biopsy, partial resection, subtotal resection, near-total resection, complete resection, and supramaximal resection (Table 2). Additionally, emerging evidence suggests that absolute residual tumor volume may be a more critical prognostic indicator than the percentage of tumor removed (114). Hence, the concept of supratotal resection (SpTR) has emerged within HGG surgery. A recent meta-analysis (2023) (115) evaluating the association between SpTR and survival outcomes in glioblastoma patients found that SpTR was associated with significantly increased OS. In certain anatomical regions, more extensive resection by means of a lobectomy may be feasible, with a meta-analysis (2023) (116) revealing that anterior temporal, frontal, or occipital lobectomy was associated with significantly better OS and PFS than GTR, but not KPS, with no significant difference in complication rates between lobectomy and GTR. Nonetheless, decision-making regarding the extent of resection must be carefully individualized, particularly in older or medically frail patients.

**Table 2:** The Response Assessment in Neuro-Oncology (RANO) criteria for extent of resection in adult diffuse high-grade gliomas.

Categories		Class	Definition	Median overall survival (months)*
Supramaximal CE resection		1	0 cm^3^ CE + ≤5 cm^3^ nCE	24 (95% CI 20–41)
Maximal CE resection	Complete	2a	0 cm^3^ CE + > 5 cm^3^ nCE	19 (95% CI 17–20)
	Near-total	2b	≤1cm^3^ nCE	
Submaximal CE resection	Subtotal	3a	≤5cm^3^ nCE	15 (95% CI 12–17)
	Partial	3b	>5cm^3^ nCE	
Biopsy		4	No reduction of tumor volume	10 (95% CI 8–12)

CE, Contrast-Enhancing tumor; nCE, non-Contrast-Enhancing tumor. *Median Overall Survival in IDH-wildtype glioblastomas treated per EORTC-26981/22981-protocol (*n* = 744, *p* = 0.001; Karschnia et al. Neuro Oncol. 2023).

#### 3.2.2 Anatomical localization of tumor

Despite the consistent survival advantage conferred by greater extent of resection, its achievement is inherently constrained by the tumor’s anatomical context. Anatomical localization plays a critical role in prognosis, as it informs the feasibility of resection and the potential for region-specific functional impairment. Tumors situated in central brain regions, such as the basal ganglia, corpus callosum, and periventricular white matter, are associated with significantly worse survival outcomes due to their limited surgical accessibility and proximity to critical neural pathways (117). The prognostic relevance of hemispheric lateralization is more nuanced and requires careful differentiation between anatomical laterality and functional dominance. Evidence suggests that tumors in the dominant hemisphere are more likely to result in postoperative neurocognitive decline, particularly when located in language-associated regions such as the left temporal lobe (117, 118). This functional burden may contribute to lower performance status, which may limit eligibility for adjuvant therapies and negatively impact clinical outcomes. Nonetheless, laterality-specific survival differences have also been observed with subregional variations within the temporal lobe influencing survival. In a population-based voxel-wise tumor atlas, Fyllingen et al. (117) demonstrated that gliomas in the left temporal pole confer a median survival <6 months, whereash those in the dorsomedial right temporal lobe have been associated with prolonged survival >24 months. Additionally, gliomas affecting the parietal lobe and lateral ventricles have been identified as markers of poor prognosis. Interestingly, emerging evidence suggests that differences in gene expression between brain hemispheres may modulate survival by generating location-dependent variations in biomarkers associated with OS (119).

Beyond cerebral hemispheric considerations, tumor localization within deep or infratentorial structures introduces additional prognostic and therapeutic challenges. Brainstem gliomas carry a particularly poor prognosis and are rarely amenable to significant surgical resection due to their involvement in vital autonomic and motor pathways, with biopsy and palliative management being the predominant treatment strategies (120). Similarly, thalamic gliomas are often managed with biopsy followed by chemoradiotherapy due to their deep-seated location and extensive integration with sensory and motor relay pathways (121). Cerebellar glioblastomas, although relatively uncommon, exhibit heterogeneous prognoses depending on their proximity to the brainstem and deep cerebellar nuclei. While some cerebellar tumors may be amenable to resection, those involving the fourth ventricle or brainstem structures are typically associated with poor survival outcomes (120). Moreover, multifocal glioblastomas, defined as glioblastomas with multiple lesions either connected via pathways of expansion or occurring independently, present unique challenges. These tumors are often associated with poorer prognosis due to their diffuse nature, involvement of eloquent or deep cerebral regions, and limited surgical resectability.

### 3.3 Systemic and metabolic prognostic modifiers

#### 3.3.1 Dexamethasone use

Dexamethasone, a potent synthetic corticosteroid, is frequently used in glioma management to reduce cerebral edema, and its associated mass effect and neurological dysfunction (14). Dexamethasone downregulates vascular endothelial growth factor (VEGF), decreasing blood-brain barrier (BBB) permeability, and upregulates calcium-activated K + channels, enhancing drug penetration into the brain (122). This steroid is commonly administered pre- and post-operatively, as well as during radiotherapy to alleviate neurological symptoms like headache, nausea, and vomiting, making it the steroid of choice in neuro-oncology due to its high potency, extended half-life, and effective brain penetration. However, recent research has raised concerns about dexamethasone’s impact on survival outcomes in glioblastoma patients. A meta-analysis (2024) (123) reported significantly poorer OS and PFS in glioblastoma patients on pre- or peri-operative dexamethasone. This meta-analysis included seven studies, with all but one accounting for key confounders such as age, KPS, extent of resection, and treatment variables such as chemotherapy and radiotherapy. This association suggests that while dexamethasone provides symptomatic relief, its use may adversely affect long-term outcomes. The impact of dexamethasone on tumor biology is complex; it has been shown to influence cellular proliferation and migration (124), with recent studies indicating that dexamethasone may facilitate glioblastoma cell migration (124), contrasting with earlier findings of anti-proliferative effects. Furthermore, dexamethasone can enhance the effects of chemotherapeutic agents like carboplatin and gemcitabine but appears to reduce the efficacy of TMZ, the standard chemotherapy for glioblastoma (125). A recent in-vitro study (126) utilized two human glioblastoma cell lines (MZ54 and U251) and found that the addition of dexamethasone significantly reduced the efficacy of RT in U251, but not in MZ54 cells. This same study utilized TTFields to induce massive cell death in both cell lines, and found no reduction in TTFields efficacy when combined with dexamethasone. These findings were further supported by a retrospective translational analysis (126), that demonstrated dexamethasone had no impact on PFS or OS in TTFields-treated patients. Of note, a meta-analysis (2022) (127) of dexamethasone use and its influence on TTFields efficacy in glioblastoma revealed that the median OS was longer in the TTFields group where the dose of dexamethasone was ≤4.1 mg (*p* < 0.05), suggesting a potential dose-dependent effect. Nonetheless, dexamethasone’s immunosuppressive properties, including suppression of both cellular and humoral immunity, likely increases susceptibility to infections and may impair immune-mediated tumor control. Furthermore, dexamethasone-induced hyperglycemia, leukocytosis, and myopathy exacerbate morbidity and are associated with poorer survival outcomes.

#### 3.3.2 Hyperglycemia

Hyperglycemia is increasingly recognized as a negative prognostic factor in HGGs (128). This condition often arises as a side effect of glucocorticoid therapy, commonly used to reduce cerebral edema, or as a result of the physiological stress response associated with severe illness (129). Elevated blood glucose levels provide an accessible energy source for cancer cells, facilitating glycolytic and oxidative phosphorylation pathways that support tumor proliferation, invasion, and survival. Hyperglycemia also drives increased lactate production, acidifying the tumor microenvironment, which enhances immune evasion and promotes resistance to treatment. Several studies have correlated hyperglycemia with diminished OS and PFS in glioblastoma patients (128). More specifically, hyperglycemia has also been shown to compromise the effectiveness of both chemotherapy and radiotherapy in HGGs patients. Elevated glucose levels alter cellular redox states and metabolic pathways, potentially reducing the cytotoxic effects of TMZ (130). Hyperglycemia is also implicated in enhancing radiation resistance (131), as high glucose concentrations can modulate oxidative stress responses and promote DNA repair mechanisms that counteract the effects of radiation-induced DNA damage. These findings suggest that hyperglycemia not only exacerbates the biological aggressiveness of gliomas but also contributes to treatment resistance; however, further research is required to fully understand the intricate impact of hyperglycemia on oncological outcomes.

#### 3.3.3 Inflammatory markers

Adding to the complexity of the glioblastoma microenvironment, inflammation plays a pivotal role in tumor progression and immune evasion (132). Systemic inflammation is a hallmark of tumorigenesis and supports all cancer stages, from initiation to metastasis. Aberrant inflammatory responses in gliomas contribute to immune tolerance, allowing tumor cells to evade therapeutic interventions, highlighting the interplay between systemic health and treatment outcomes. Notably, glioblastoma distinguishes itself from other gliomas by its pronounced ability to cultivate a highly inflammatory and immune-suppressed environment, fostering an aggressive, treatment-resistant phenotype capable of evading immune surveillance (132). Preoperative systemic inflammatory responses, coagulation function, and nutritional status significantly influence antitumor efficacy in glioma patients, emphasizing the intricate interplay between systemic health and treatment outcomes. C-reactive protein (CRP), a key marker of systemic inflammation, is strongly associated with advanced tumor stage, therapy resistance, and poorer survival outcomes in HGGs (133). Moreover, several inflammatory markers, including the neutrophil-to-lymphocyte ratio (NLR), platelet-to-lymphocyte ratio (PLR), lymphocyte-to-monocyte ratio (LMR), red cell distribution width (RDW), systemic immune-inflammation index (SII), and systemic inflammation response index (SIRI), have been investigated as prognostic indicators in cancer, during the entire peri-operative period (134). A recent meta-analysis (2023) (135) highlighted the prognostic significance of NLR and PLR, with NLR emerging as a key predictor that may guide chemotherapy modifications for high-risk patients. Interestingly, recent integrative evidence reinforces these associations. In a large cohort of 176 glioblastoma patients, Asey et al. (136) demonstrated that elevated peripheral neutrophil counts were independently associated with significantly shorter OS (median 10 vs. 17 months; *p* = 0.01), whereas other immune cell ratios such as NLR and PLR did not retain prognostic power when dichotomized by the median. Importantly, this study also revealed dynamic changes in immune cell populations over disease progression: at first recurrence, lymphocytes, monocytes, neutrophils, and platelets were all decreased, yet elevated monocyte, neutrophil, and platelet counts at recurrence correlated with poorer survival outcomes. When stratified by DNA methylation subclass, distinct immunological patterns emerged. Within the mesenchymal (MES) glioblastoma subtype, characterized by heightened immune activity and inflammatory signaling, both higher neutrophil and lower lymphocyte counts were linked to worse outcomes (median OS 14 vs. 22 months; *p* = 0.007), whereas the receptor tyrosine kinase (RTK) I and II subtypes showed weaker or no associations. Deconvolution analyses of matched tumor tissue further revealed that circulating platelet and monocyte levels correlated with tumor tissue signatures reflecting a more differentiated, tumor-progressive cell state, while peripheral immune profiles were most accurately mirrored in MES tumors. Of note, glioblastoma induces not only quantitative but also qualitative immune dysfunction, characterized by systemic immune anergy noticeable by lymphopenia and reduced CD4+/CD8 + T-cell subsets; a state of peripheral immune paralysis marked by impaired T-cell activation, expansion of myeloid-derived suppressor cells, and functional exhaustion. This paradoxical coexistence of inflammation and immune suppression contributes to lymphopenia, elevated NLR, and diminished antitumor immunity, ultimately promoting therapeutic resistance and poor survival (137).

Collectively, these findings suggest that systemic inflammation in glioblastoma reflects both tumor-intrinsic biology and host immune status. Elevated neutrophil and platelet counts may signify a shift toward a pro-tumor inflammatory milieu promoting angiogenesis, immune suppression, and resistance to cytotoxic therapy. Furthermore, the integration of hematologic markers with molecular subclassification enhances the predictive resolution of systemic inflammatory indices. This interplay between systemic inflammation and molecular subtype underscores the need for stratified biomarker frameworks that capture both peripheral immune dynamics and intrinsic tumor epigenetic states to guide individualized prognostication and therapy selection in glioblastoma.

#### 3.3.4 Circulating and systemic biomarkers

Systemic circulating biomarkers, including cell-free DNA (cfDNA), tumor-derived extracellular vesicles (EVs), and peripheral immune cell profiles, have emerged as promising, minimally invasive tools for dynamic prognostication in HGGs. cfDNA, released into circulation through apoptosis, necrosis, active secretion, or neutrophil extracellular trap formation (NETosis), reflects real-time tumor burden and genomic evolution (138). Quantitative and mutational analyses of cfDNA have demonstrated significant associations with OS and disease progression (139, 140), with elevated cfDNA concentrations and detectable tumor-specific mutations correlating with poorer prognosis. Moreover, longitudinal cfDNA monitoring can identify recurrence several months before radiographic progression, underscoring its potential as an early indicator of treatment resistance and minimal residual disease. Similarly, EVs, including exosomes and microvesicles, serve as another critical source of tumor-derived nucleic acids, proteins, and metabolites that faithfully mirror intratumoral molecular states (141). Elevated plasma concentrations of glioma-derived EVs have been linked to higher WHO grade, increased angiogenic signaling, and reduced OS (141, 142). In addition, EV-associated MGMT mRNA and microRNA signatures, particularly miR-21 and miR-222, have been correlated with chemoresistance and unfavorable clinical outcomes, suggesting a predictive role for EV profiling in assessing TMZ responsiveness (143). Collectively, these circulating biomarkers provide a window into tumor dynamics and systemic response, offering significant potential for integration into multimodal prognostic frameworks that bridge molecular pathology and clinical surveillance in HGGs.

#### 3.3.5 Nutritional status and immune function

Nutritional status, assessed through metrics such as the prognostic nutritional index (PNI) and serum albumin levels, is widely acknowledged for its prognostic importance. Reduced PNI and serum albumin levels consistently correlate with increased tumor aggressiveness, compromised immune function, and diminished survival rates. The global immune-nutrition-inflammation index (GINI), a composite metric combining immune, nutritional, and inflammatory parameters, has emerged as a promising prognostic tool in gliomas (144), as low GINI scores are associated with adverse outcomes, capturing the synergistic effects of inflammation and malnutrition on immune competence and overall prognosis.

### 3.4 Health system and sociodemographic disparities

#### 3.4.1 Socioeconomic determinants of prognosis

Socioeconomic factors significantly influence survival outcomes in HGGs, particularly through complex interactions involving healthcare access, treatment disparities, and broader social determinants of health (SDoH). A meta-analysis (2024) (145) of 143,303 glioblastoma patients revealed significantly worse survival outcomes for individuals with lower socio-economic status (SES). Specifically, studies underscore the impact of lower SES (145) and higher scores on the area deprivation index (ADI) (146) on access to and quality of treatment. Rivera Perla et al. (146) demonstrated significantly lower rates of GTR, reduced odds of receiving chemoradiation, and decreased access to clinical trials among socioeconomically disadvantaged patients. Similarly, Pollom et al. (147) found that individuals from higher-income neighborhoods in California were significantly more likely to receive radiation therapy within 35 days of GTR, with delays in radiation therapy initiation being strongly associated with inferior outcomes. This pattern is mirrored in studies of systemic chemotherapy (148), analyzing 16,682 glioblastoma patients in the SEER database, which revealed that increased household income significantly improved the likelihood of receiving systemic chemotherapy. Noteworthy, SES disparities persist even in universal healthcare systems (149).

#### 3.4.2 Geographic disparities and the influence of high-volume Centers on care access and clinical outcomes

Geographic location amplifies SES-related inequities (150), with urban patients generally benefiting from proximity to high-volume academic centers that offer multidisciplinary, multimodal care. In contrast, rural patients face logistical challenges, including extended travel distances, limited access to specialized facilities, and delays in receiving essential treatments like surgical resection and radiation therapy (151), resulting in shorter median survival. Adherence to treatment is crucial for maximizing survival in glioblastoma patients, with an analysis (131) of 17,451 cases from the *National Cancer Database* (NCDB) showing a strong correlation between completing conventionally fractionated chemoradiotherapy and improved outcomes. Patients completing ≥58 Gy had a median OS of 13.5 months, compared to near-completers (50–58 Gy; median OS of 5.7 months) and non-completers (<50 Gy; median OS of 1.9 months) (*p* < 0.001). Non-completion of therapy was disproportionately observed among patients treated at low-volume centers. Similar patterns have been reported globally, where urban patients demonstrate better survival outcomes due to the unequal distribution of healthcare resources. Interestingly, tumor aggressiveness plays a notable role in shaping these dynamics. For indolent tumors, such as oligodendrogliomas, socioeconomic differences are amplified as patients with greater resources often have access to a wider array of treatment options and prolonged care. In contrast, the highly aggressive nature of glioblastoma may mitigate the impact of socioeconomic and geographic disparities, as the critical need for immediate treatment often prioritizes access regardless of background.

#### 3.4.3 Racial and ethnic disparities in treatment and survival

Racial and ethnic disparities in glioblastoma survival have been extensively examined through large-scale studies (152), revealing significant differences in incidence, access to treatment, and survival outcomes across diverse populations. Epidemiological data (153) indicate that survival rates vary by race, with Asian patients frequently achieving the highest five-year survival rates, Black and Hispanic patients exhibiting intermediate survival rates (154), and White patients experiencing the lowest survival outcomes. However, the interpretation of these disparities remains complex due to the multifaceted interplay between race and broader SDoH. Indeed, Ostrom et al. (153) reported that Black and Hispanic patients were significantly less likely to receive radiation and chemotherapy than White patients, and also experienced longer delays in treatment initiation. Nonetheless, even after adjusting for known prognostic factors and treatment characteristics, race and ethnicity remained independently associated with survival outcomes (153). Further, Liu et al. (155) demonstrated that racial background influences glioblastoma-associated mortality independently of tumor biology and treatment patterns, while also contributing to non-glioblastoma mortality, including deaths from other cancers and cardiovascular events. Beyond disparities in treatment access and timing, emerging evidence suggests that racial differences in glioblastoma survival may also have genetic and molecular underpinnings (156). These findings highlight not only the potential influence of race on tumor genomics but also the clinical significance of racial disparities in actionable genetic alterations. Despite these insights, a significant limitation of the current literature on racial disparities in glioblastoma is the reliance on database studies that primarily use self-reported race. Self-reported racial categories may not accurately reflect an individual’s genetic ancestry, as racial classification is often based on phenotypic characteristics and sociopolitical constructs rather than true genetic lineage (157).

## 4 Tumor recurrence and grade progression

In diffuse IDH-mutant lower-grade gliomas (LGGs), malignant relapse and grade progression are dictated by an interplay of clinical, radiographic, and molecular determinants. Surgical series (158, 159) consistently show that gross-total resection, often defined as ≥90–100% resection of the T2/FLAIR volume, and minimal postoperative residual disease correlate with substantially longer time to progression and reduced risk of high-grade transformation, whereas early recurrence within approximately 2 years of diagnosis, rapid radiographic growth, or non-local (multifocal or distant) relapse strongly predict malignant evolution and poor post-recurrence survival. On MRI, the development or enlargement of contrast enhancement, particularly when associated with restricted diffusion reflecting low apparent diffusion coefficient (ADC) values and high cellularity, serves as a sensitive indicator of anaplastic change, while non-enhancing, radiographically stable lesions generally follow a more indolent course (160). At the molecular level, IDH-mutant astrocytic LGGs typically harbor *TP53* mutations and *ATRX* loss at baseline, but progression to grade 3/4 disease is driven by additional “late” genetic alterations, most notably bi-allelic *CDKN2A*/*B* deletion, which now defines a grade 4 IDH-mutant astrocytoma under the WHO CNS5 classification even in the absence of necrosis, and which is highly enriched at recurrence with sharply adverse prognostic impact; even hemizygous *CDKN2A* loss independently portends shorter OS in recurrent non-codeleted gliomas (161, 162). Treatment-induced changes also contribute, as TMZ exposure may generate a hypermutator phenotype through mismatch repair (MMR) deficiency, and recurrent LGGs acquiring this signature almost invariably transform to high-grade tumors, recur distantly, and demonstrate markedly shortened survival (150, 163).

## 5 Integrated prognostic framework and future directions

### 5.1 Radiomics, radiogenomics, and AI in structural imaging

Structural neuroimaging constitutes a fundamental pillar of prognostication in HGGs (151). Pre-operative tumor size, as determined through MRI, has been widely studied as a prognostic factor in glioblastoma, with several retrospective reviews identifying larger tumoral diameter as associated with inferior OS (152). Different size cutoffs for significant prognostic impact have been proposed (ranging from 4 to 6 cm), reflecting variability across studies (153). Initially, the Macdonald criteria recommended two-dimensional (2D) diameters which relies on cross-sectional imaging to gage tumor dimensions (154) for evaluating therapeutic response (155), an approach subsequently endorsed by the *Response Assessment in Neuro-Oncology* (RANO) group. However, the irregular morphology characteristic of HGGs can challenge the accuracy of both 2D and 3D ellipsoid protocols, often limiting precise measurement. In recent years, advancements in imaging have introduced semi-automatic segmentation algorithms within 3D image-processing software, which improve the accuracy of volumetric measurements, particularly for tumors with irregular shapes (156). These tools enable clinicians to delineate distinct tumor compartments, including necrotic areas, contrast-enhancing regions, and FLAIR hyperintense volumes. The prognostic relevance of these segmented volumes varies. Large FLAIR hyperintense volumes, indicative of peritumoral edema and often infiltrative tumor cells, is generally associated with poorer prognosis (157, 164). However, the relationship between FLAIR hyperintensity and OS remains complex (165, 166); some studies indicate a positive correlation, while others report no significant impact on survival. Additionally, necrotic volume, contrast-enhancing tumor volume, and the tumor-to-necrosis volume ratio have each been correlated with survival outcomes, though results are inconsistent across studies (165, 166). High necrotic volume may reflect tumor hypoxia and aggressive cellular turnover, both of which are markers of malignancy and correlate with poorer outcomes (166). Similarly, radiomics analysis of cerebral blood flow (CBF) has suggested that perfusion homogeneity, as indicated by features such as Zone Size Variance (ZSV) and Correlation, can provide additional prognostic insights, with higher homogeneity correlating with poorer survival outcomes (167). The enhancement volume is another critical measure, as larger enhancing volumes suggest active tumor regions with higher cellular proliferation and angiogenesis, factors often associated with adverse prognosis (152). Hence, the integration of advanced imaging techniques, particularly volumetric segmentation and radiomics-based perfusion analysis, continues to refine prognostic assessments by offering more precise tumor characterization and refinements for surgical planning.

Radiogenomics extends these principles by mapping imaging phenotypes onto underlying molecular alterations (151). Multiparametric MRI signatures have demonstrated predictive value for mutations in *EGFR*, *TP53*, *PTEN*, and *NF1*, as well as pathway-level aberrations in *RTK*, *PI3K*, and *MAPK* signaling cascades. Such associations are biologically coherent: *EGFR* amplification correlates with elevated cerebral blood volume and poor survival, while *TP53* mutations are linked to increased permeability and infiltrative morphology (168). The T2–FLAIR mismatch sign exemplifies a highly specific radiogenomic biomarker of IDH-mutant astrocytomas, conferring strong prognostic value even in the absence of histological confirmation (151).

The integration of artificial intelligence (AI) has accelerated the translational potential of radiomics and radiogenomics. Machine learning workflows, encompassing tumor segmentation, high-throughput feature extraction, and predictive modeling, have consistently outperformed traditional clinical predictors of PFS and OS. Deep learning frameworks trained on conventional MRI can infer molecular features of direct prognostic relevance (169, 170). Furthermore, hybrid modalities combining amino-acid PET with MRI radiomics surpass 0.85 in predicting IDH and 1p/19q status, directly linking molecular inference with survival stratification (171, 172). Radiogenomics epitomizes a paradigm shift from static, morphology-based assessment toward dynamic, image-informed molecular and prognostic inference. By generating “virtual genotypes” and survival indices from entire tumor volumes, imaging transcends its diagnostic role to serve as a noninvasive molecular assay and prognostic tool.

### 5.2 Integration of AI and future directions in HGGs prognostication

Artificial intelligence is increasingly shaping the broader neuro-oncology landscape by extending prognostication, workflow automation, and treatment planning. Automated segmentation platforms, such as the FDA-cleared Neosoma HGG, now enable accurate volumetry and longitudinal tracking, directly supporting clinical decision-making. Beyond imaging, “pathomics” applies deep learning to digitized histopathology, predicting IDH status with area under the curve (AUC) >0.90 and stratifying risk with prognostic indices approaching 0.74 (173). Multimodal frameworks (174, 175) that integrate radiology, pathology, and molecular data reveal novel glioma subtypes with distinct biological signatures and therapeutic sensitivities, pushing beyond current WHO classifications. Indeed, advances in computational methodologies are revolutionizing prognostication and treatment planning in HGGs by identifying complex patterns in multi-dimensional datasets. Machine learning models, integrating clinical, imaging, and molecular data, have achieved high predictive accuracy (AUC 0.80–0.95) (176) for survival, recurrence, and treatment response, though challenges remain in standardizing data acquisition, external validation, and ensuring model interpretability.

### 5.3 Proposed integration framework and implementation strategies

Accurate prognostication in HGGs demands the convergence of biological, clinical, therapeutic, and contextual determinants into unified models capable of informing individualized care. We have outlined a framework of prognostic and predictive factors under four main domains: (1) tumor-intrinsic biology; (2) patient-level variables; and (3) system-level health disparities modifiers (Figure 1). These domains have components that may change over the treatment journey of a particular patient, both in time and space, requiring multi-dimensional analysis.

Despite major advances in molecular neuro-oncology, the datasets underpinning current prognostic models remain comparatively constrained. Routine diagnostic testing typically encompasses only canonical alterations, most commonly *IDH* mutation and *MGMT* promoter methylation, while omitting a wealth of potential biological and immunological parameters. Systematic integration of additional variables, such as peripheral immune-cell ratios, tumor-infiltrating lymphocyte composition, and checkpoint-molecule expression (e.g., PD-1, PD-L1, and CTLA-4), would markedly enhance dataset granularity and biological interpretability. Incorporating such metrics into standardized biobanking pipelines and electronic medical record (EMR) systems could enable the generation of high-resolution, multimodal datasets linking tumor biology with host immunity, treatment response, and outcomes.

Computational platforms provide a promising means of harmonizing these multimodal inputs into clinically interpretable prognostic indices (177, 178). Future iterations, however, must move beyond strictly biological variables to incorporate systemic and contextual determinants, ensuring that predictions are not only precise but also equitable. This evolution from siloed variables to holistic, multidimensional prognostic models represents an essential step toward precision medicine and health equity in neuro-oncology (179). Translating this framework into practice requires deliberate integration with clinical workflows. Embedding structured fields for core prognostic variables into EMRs would permit automated risk estimation at the point of care. Linked calculators could generate composite prognostic scores from molecular and sociodemographic data, facilitating real-time use in clinic visits and tumor board discussions. AI–based tools extend this capacity, with platforms already providing automated volumetry and longitudinal tracking, while deep-learning models can infer key molecular alterations from MRI with high accuracy. Integration of such tools into EMRs could enable automated risk flagging, guiding surveillance and treatment planning.

Moreover, effective adoption requires workflow alignment. Structured checklists and standardized templates at multidisciplinary tumor boards can ensure consistent integration of all four prognostic domains (180, 181), while automated reports synthesizing imaging, molecular, and contextual data can provide reproducible decision support. Sustained clinician training, iterative feedback, and linkage to quality metrics, such as treatment timeliness or equitable enrollment of underserved patients, will be critical to reinforcing the systematic consideration of non-biological determinants. The clinical utility of this framework lies in its ability to personalize therapy. Patients with unfavorable tumor biology and poor performance status may be counseled toward clinical trial enrollment or early palliative integration, whereas favorable-risk patients may be directed toward maximal therapy with long-term surveillance (182). Socioeconomic barriers identified through the framework can prompt early referral to social services or care coordinators, mitigating risks of non-adherence. The framework can enrich patient counseling by providing individualized survival estimates and emphasizing modifiable risk factors, such as hyperglycemia, malnutrition, or systemic inflammation, that may be targeted through supportive interventions. At the policy level, aggregated framework-derived data can reveal population-level disparities, thereby guiding targeted interventions including telemedicine outreach, subsidized care programs, and strategic resource allocation (e.g., nutritionists, patient navigation).

Beyond clinical care, systematic and structured data acquisition holds significant implications for translational research and clinical-trial design. Enriched datasets encompassing molecular, immune, and contextual features can refine patient stratification, improving the linkage between predictive biomarkers and therapeutic responsiveness. Inadequate biological stratification and heterogeneous patient selection are recurrent limitations in neuro-oncology trials; a more granular understanding of prognostic and predictive determinants would facilitate rational allocation of novel therapies to biologically defined subgroups, enhancing both efficacy and trial interpretability.

In sum, embedding this multidimensional prognostic framework within EMR workflows, AI-enabled decision support systems, and health-policy initiatives offers a feasible and scalable pathway for translating prognostic science into equitable, patient-centered neuro-oncology care.

## 6 Conclusion

HGGs represent a biologically heterogeneous group of malignancies, in which tumor-intrinsic factors constitute the principal determinants of prognosis, therapeutic response, and disease progression (Table 3). The incorporation of molecular diagnostics has redefined the classification of HGGs, enabling more precise prognostic stratification and uncovering biologically distinct subtypes with divergent clinical trajectories. Meanwhile, emerging insights into molecular pathways, the tumor microenvironment, immune landscape, and radiogenomic features are further refining our understanding of glioma biology and resistance mechanisms. These discoveries not only deepen prognostic modeling but also inform the development of rational, targeted therapies aimed at exploiting tumor-specific vulnerabilities. The advent of computational tools and integrative multi-omics approaches now provide the opportunity to synthesize these complex data into more of increasingly accurate and individualized prognostic models. However, the survival of HGGs is also determined by a multifaceted interplay of clinical, therapeutic, and systemic factors (Figure 1). Recognizing the prognostic and predictive relevance of these non-tumor factors underscores the need for an integrative framework that incorporates patient and system-level determinants. Such a model is essential not only for advancing precision oncology but also for guiding equitable clinical decision-making and health policy reform in the care of patients with HGGs.

**Table 3:** Integrated overview of prognostic and predictive factors in high-grade gliomas.

Category	Biomarker/Feature	Prognostic role	Predictive role	Rationale	Note
Tumor-intrinsic molecular and histopathological factors	*IDH* mutation (IDH1/2)	*Positive*	Predicts sensitivity to IDH inhibitors	Mutant IDH produces 2-hydroxyglutarate, reprogramming metabolism/epigenetics to a less aggressive state.	IDH-R132H is present in ~90% of IDH-mut gliomas. Non–R132H IDH mutations linked to even better outcomes.
	*MGMT* promoter methylation	*Positive*	Strong predictor of benefit from alkylating chemo (TMZ)	MGMT silencing prevents DNA repair of TMZ-induced lesions, making cells more chemosensitive.	Key stratifier in elderly GBM; degree of methylation and epigenetic subgroup can modulate its impact.
	*TERT* promoter mutation	*Mixed*	—	Enables telomere maintenance and cellular immortality.	Combined MGMT-met+TERT-mut gave longest OS in one study, but results vary.
	*EGFR* amplification (*EGFRvIII*)	*Negative*	— (EGFR inhibitors have failed in trials)	Drives proliferation and invasion via PI3K/AKT signaling; EGFRvIII is constitutively active.	Part of chr7 gain; therapies (TKIs, vaccines) have shown limited efficacy.
	*PTEN* deletion/mutation	*Negative*	—	PTEN loss (often via chr10 deletion) removes an inhibitory brake on growth pathways.	Often co-occurs with EGFR amp; PTEN status is a poor prognostic marker.
	*CDKN2A*/*B* homozygous deletion	*Negative* in IDH-mut astrocytoma	—	Loss of tumor suppressors p16/p14 leads to unchecked cell cycling.	In WHO CNS5, any IDH-mut astro with CDKN2A/B deletion is grade 4. In oligodendroglioma, it marks an aggressive subset.
	*ATRX* loss/mutation	*Positive* IDH-mut astrocytoma	Predicts sensitivity to PARP inhibitors and DNA damage therapies	ATRX loss causes telomere instability and DNA repair defects, creating vulnerabilities (synthetic lethality).	Mutually exclusive with 1p/19q codeletion; used diagnostically (ATRX IHC) to confirm astrocytoma subtype.
	*TP53* mutation	*Positive* in IDH-mut gliomas	—	Loss of p53 causes instability but in IDH-mut context indicates a relatively indolent biology.	Absent in 1p/19q-codeleted tumors; not specific in GBM context.
	1p/19q codeletion	*Positive* in IDH-mut oligodendroglioma	Predicts response to combined RT + PCV chemotherapy	Defines oligodendroglioma lineage (ATRX intact, TERT-mut); these tumors are chemo-sensitive.	Required for oligodendroglioma diagnosis; rare cases with CDKN2A/B loss have worse prognosis.
	Autophagy-related signature	*Negative*	High autophagy may predict benefit from autophagy inhibitors	Autophagy provides survival mechanism under stress (hypoxia, chemo).	Clinical trials of HCQ ± CRT show promise in some, but not all patient groups.
Radiogenomic and imaging	Tumor size	*Negative*	—	Larger tumors imply higher tumor burden; hard to resect completely.	Various studies use 4–6 cm cutoffs; 3D volumetric methods improve measurement accuracy.
	FLAIR hyperintensity volume	*Negative*	—	Reflects peritumoral edema and invasive tumor cells; more extensive spread worsens prognosis.	Relationship to OS is complex (some studies disagree).
	Necrotic tumor volume	*Negative*	—	Necrosis indicates hypoxia and rapid growth (malignancy).	Measured as absolute volume or ratio; high necrosis is a marker of aggressiveness.
	Enhancing volume	*Negative*	—	Enhancing region reflects proliferative, angiogenic tumor.	Used in segmentation (e.g., enhancing:necrotic ratio); correlates with tumor grade.
Tumor microenvironment and immune factors	TAMs – M2	*Negative*	Under investigation (CSF1R inhibitors)	M2-like TAMs produce IL-10, TGFβ, VEGF that suppress immunity and promote angiogenesis.	TAMs can be 30–50% of GBM mass; enriched in mesenchymal subtype.
	Tregs	*Negative*	—	Tregs secrete inhibitory cytokines, dampening cytotoxic T-cell response.	Elevated intratumoral Tregs are a hallmark of immunosuppression in GBM.
	MDSCs	*Negative*	—	MDSCs inhibit antigen presentation and T/NK-cell activity.	Found in blood and tumor; blockade of MDSCs is an active research area.
	PD-1/PD-L1 expression	*Negative*	Under investigation (checkpoint inhibitors)	Immune checkpoints inhibit T-cell function; high PD-L1 indicates an exhausted immune milieu.	Assay variability is high; still, PD-L1 ≥ in high-grade tumors of advanced stage.
	CTLA-4 expression	*Negative*	Under investigation (checkpoint inhibitors)	CTLA-4 suppresses T-cell priming; elevated in glioma TME contributes to immune escape.	Often co-expressed with PD-1/PD-L1; high levels imply immunosuppression.
	CD44 expression	*Negative*	—	CD44 mediates cell adhesion/migration and is associated with TAM recruitment and mesenchymal transition.	Marker of aggressive, invasive phenotype; linked to extracellular matrix interaction.
	MARCO expression	*Negative*	—	MARCO is a scavenger receptor on macrophages, marking a pro-tumor subset.	Emerging biomarker from transcriptome studies of mesenchymal GBM.
	CD47–SIRPα axis	*Negative*	Under investigation (anti-CD47 agents)	CD47 on tumor binds SIRPα on macrophages, inhibiting phagocytosis.	Preclinical blockade enhances clearance; clinical efficacy under investigation.
	TREM2 expression	*Unclear*	—	TREM2 on myeloid cells may indicate an immune-active microenvironment.	Early data suggest it marks a specific immune state; prognostic impact not yet proven.
	ctDNA	*Negative*	Under investigation (TMZ-induced MMR mutations)	ctDNA in plasma/CSF reflects tumor burden; serial monitoring can identify recurrence or resistance earlier.	ctDNA detection in GBM is challenging (low amounts), but positive findings predict progression.
	cfDNA	*Negative*	—	cfDNA (from NETosis/apoptosis) reflects tumor/inflammation.	cfDNA as a glioma biomarker is emerging.
Treatment-related factors	High EOR	*Positive*	—	More resection removes aggressive cells; minimal residual disease delays recurrence.	RANO criteria now include %EOR; “supratotal” (beyond enhancement) improves OS further.
	Residual tumor volume	*Negative*	—	Residual mass acts as nidus for recurrence; absolute residual may trump % resection.	Emphasis shifting to minimizing absolute residual volume.
	Tumor location (Deep or central) (basal ganglia, corpus callosum, periventricular) and multifocal	*Negative*	—	Limits safe resection; involvement of eloquent areas lowers functional status.	-
	Treatment adherence	*Positive*	—	Full protocol maximizes tumor management; incomplete treatment (due to delay or cessation) sharply reduces survival.	NCDB analysis: median OS dropped from 13.5 mo (≥58 Gy) to 1.9 mo (<50 Gy).
	Dexamethasone use	*Negative*	Reduces TMZ and RT efficacy in preclinical studies.	Steroid immunosuppression (lymphocyte suppression), metabolic effects (hyperglycemia), and possible pro-migratory effects on glioma cells.	Meta-analyses show worse outcomes even after adjusting for confounders.
Patient-level clinical and demographic factors	Age	*Negative*	—	Older patients have immunosenescence and less tolerance for aggressive therapy.	In IDH-mut gliomas, age has a weaker effect; very elderly (≥70) with IDH-mut tumors still do worse.
	Performance status (KPS/ECOG)	*Positive*	—	Reflects patient’s functional reserve; higher KPS enables full treatment delivery.	Post-op KPS often more prognostic than pre-op. Even small improvements in KPS translate to better outcomes.
	Sex (Female)	*Positive*	—	Sex hormones and X-linked genes (e.g., higher KDM6A in females) modulate tumor biology.	Males have higher incidence; EGFR-risk variants more in males, TERT-risk in females. Sex differences also affect treatment patterns.
	Comorbidities	*Negative*	—	Other illnesses (cardiac, diabetes, respiratory) limit treatment tolerance and options.	Frail or elderly patients often undergo less aggressive therapy for quality-of-life considerations.
Systemic and metabolic modifiers	Hyperglycemia	*Negative*	—	Tumors exploit glucose for growth (glycolysis), and high glucose promotes radiation and chemo resistance.	May exacerbate by steroids; glycemic control is important.
	High NLR/CRP	*Negative*	—	Systemic inflammation supports tumor growth; e.g. NLR (neutrophil-to-lymphocyte) is independent prognostic factor.	Meta-analysis found NLR top predictor; CRP elevation marks aggressive disease.
	High Nutritional status (PNI, albumin, GINI)	*Positive*	—	Malnutrition and immune compromise weaken host defense and therapy response.	-
Health system and sociodemographic determinants	High SES	*Positive*	—	Affects access to care: lower GTR rates, less chemo/trials in disadvantaged patients.	Even with universal healthcare, SES disparities persist.
	Geography (Rural)/Care access (low volume)	*Negative*	—	Distance delays treatment; fewer resources (surgery, radiation) available off-center.	Urban/high-volume centers confer survival advantage, especially for indolent tumors like oligodendroglioma.
	Race / Ethnicity	*Variable*	—	Likely reflects complex mix of genetics and socioeconomic factors; Black/Hispanics often under-treated.	Even adjusting for confounders, race independently affects survival. Genetic ancestry vs. self-reported race is an open question.

ATRX – Alpha Thalassemia/Mental Retardation Syndrome X-linked, CDKN2A/B – Cyclin-Dependent Kinase Inhibitor 2A/2B, CFDNA – Cell-Free DNA, CRT – Chemoradiotherapy, CRP – C-Reactive Protein, CTLA-4 – Cytotoxic T-Lymphocyte Antigen-4, ctDNA – Circulating Tumor DNA, ECOG – Eastern Cooperative Oncology Group (performance status), EGFR – Epidermal Growth Factor Receptor, EGFRvIII – EGFR Variant III (mutant isoform), EOR – Extent of Resection, FLAIR – Fluid-Attenuated Inversion Recovery, GBM – Glioblastoma Multiforme, GINI – Geriatric Nutritional Index, GTR – Gross Total Resection, IDH – Isocitrate Dehydrogenase, IHC – Immunohistochemistry, IL-10 – Interleukin-10, KPS – Karnofsky Performance Status, MARCO – Macrophage Receptor with Collagenous Structure, MDSC – Myeloid-Derived Suppressor Cell, MGMT – O6-Methylguanine-DNA Methyltransferase, MMR – Mismatch Repair, NLR – Neutrophil-to-Lymphocyte Ratio, OS – Overall Survival, PARP – Poly(ADP-Ribose) Polymerase, PCV – Procarbazine, Lomustine (CCNU), and Vincristine, PD-1 – Programmed Death-1, PD-L1 – Programmed Death-Ligand 1, PI3K/AKT – Phosphoinositide 3-Kinase/Protein Kinase B pathway, PNI – Prognostic Nutritional Index, PTEN – Phosphatase and Tensin Homolog, RT – Radiotherapy, SES – Socioeconomic Status, SIRPα – Signal Regulatory Protein Alpha, TAM – Tumor-Associated Macrophage, TGF-β – Transforming Growth Factor Beta, TERTp – Telomerase Reverse Transcriptase promoter, TKI – Tyrosine Kinase Inhibitor, TMZ – Temozolomide, TME – Tumor Microenvironment, TP53 – Tumor Protein p53, Tregs – Regulatory T Cells, TREM2 – Triggering Receptor Expressed on Myeloid Cells 2, WHO CNS5 – World Health Organization Classification of Tumors of the Central Nervous System, 5th edition.
